# Ion-Mediated Structural Discontinuities in Phospholipid Vesicles

**DOI:** 10.1021/acs.langmuir.4c01219

**Published:** 2024-07-09

**Authors:** Judith U. De Mel, Stefanie Klisch, Sudipta Gupta, Gerald J. Schneider

**Affiliations:** † Department of Chemistry, Louisiana State University, Baton Rouge, Louisiana 70803, United States; ‡ Department of Physics and Astronomy, Louisiana State University, Baton Rouge, Louisiana 70803, United States

## Abstract

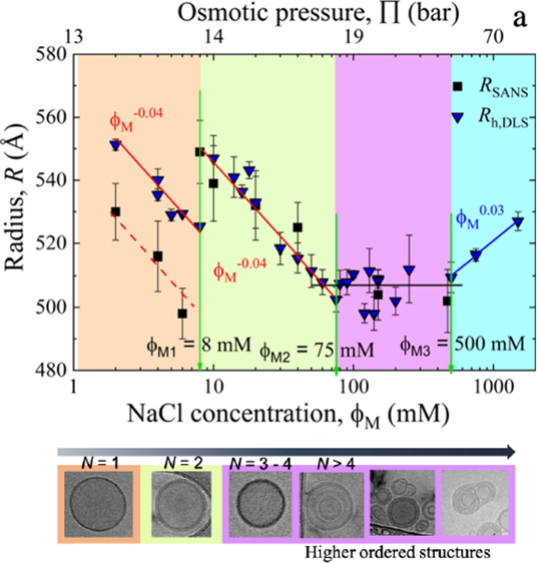

Despite intense research, methods for controlling soft matter’s spontaneous self-assembly into well-defined layers remain a significant challenge. We observed ion-induced structural discontinuities of phospholipid vesicles that can be exploited for controlled self-assembly of soft materials, using DOPC and NaCl as a model system. The observations were made for the 0.25 wt % lipid concentration. We used dynamic light scattering, zeta-potential measurement, cryo-electron microscopy, small-angle X-ray, and small-angle neutron scattering to understand the reason for the discontinuities. For salt concentrations below 8 mM, we observed a decrease in the liposome diameter with increased NaCl concentration. Above 8 mM, we measured a discontinuity; the radius increases within a very narrow salt concentration range within less than 0.1 mM and then decreases for values greater than 8 mM. At 75 mM, the radius becomes constant until it grows again at around 500 mM. Microscopy and scattering experiments show a transition from unilamellar to bilamellar at 8 mM and to trilamellar at 75 mM. At 500 mM, we found a heterogeneous liposome system with many different bilayer numbers. All the experimental observations indicate that declining solvent quality and increasing osmotic pressure direct lipids to expel preferentially to the inner compartment. Upon reaching a critical concentration, excess lipids can form a new bilayer. This spontaneous self-assembly process causes simultaneous shrinkage of the aqueous core and expansion of the vesicle. This approach opens an intriguing path for controlling the self-assembly of bioinspired colloids.

## Introduction

As much as 70% of the earth’s surface is covered by water, but 97% of all the water on earth is saline.^1^ Oceanic salt concentrations range from 400 to 450 mM, whereas freshwater sources such as rivers and lakes contain minimal salts of 0.25 mM.^2,3^ In hypersaline environments such as the Dead Sea, which is about ten times saltier than the ocean, sodium and chloride collectively amount to 78% of the salinity.^4^ Concerning the effect of salt on living cells, bacteria such as *E. coli* have a cytosolic NaCl concentration of about 5 mM, while the human cytosolic concentration ranges from 4 to 12 mM.^5−7^ Human blood contains salt in the 110–150 mM range.^7^ The tolerance of living cells to salinity differs significantly based on factors such as homeostasis mechanisms and environmental adaptations. For example, high serum sodium in humans is related to complications such as hypernatremia, cardiovascular diseases, and failures in the nervous system.^8−11^ Low serum sodium is associated with hyponatremia.^10^ Therefore, it is vital to understand the effects of extracellular salt on phospholipid membranes, particularly at the outer lipid membrane, which occupies the largest surface area of the living cells.

In this context, we focus on sodium chloride (NaCl), which is one of the most abundant salts that can be found in both biotic and abiotic environments, such as extracellular, intercellular fluids of living organisms and the earth’s oceans. Concerning biological functioning, interbilayer forces are known to be modified in the presence of monovalent salts, which affect different biological processes, like cell fusion and secretion.^12^ A number of recent studies have investigated the interactions of ions with unilamellar membranes and their effect on the vesicle structure. However, the exact nature of interactions with unilamellar membranes and the overall effect on the vesicle structure is still unclear. The presence of NaCl on the inside or the outside of the vesicle leads to structural and dynamic changes in the assembly.^13^ Claessens et al. showed the effect of a monovalent salt on the diameter of DOPC vesicles.^14^ Specifically, this study found a decrease in size above a certain salt concentration followed by an increase in diameter upon a further increase in the salt concentration. The appearance of aggregates was taken to be the reason for the size increase. A previous study by De Mel et al. indicated that NaCl can increase the vesicle’s lamellarity.^15^ The explanation may be offered by a theoretical work by Tayebi et al., who showed that while higher osmotic pressure generally results in a shrinking, vesicles with a higher membrane rigidity are more resistant to this pressure and retain larger diameters.^16^ In addition, smaller liposomes are more resistant to osmotic pressure with a larger number of bilayers.

The dipolar nature of the head groups and the dielectric gradient across the membrane interphase will contribute to ion-lipid interactions in the presence of salt. Hereafter, we concentrate on zwitterionic or “neutral” lipids with phosphatidylcholine (PC) headgroups—the most abundant phospholipids in mammalian cells.^17^ Electrically neutral PC-based membranes are known to attract one another by weak van der Waals forces due to charge fluctuations.^18,19^ The repulsion between the lipid bilayers can originate from the thermal undulation of the membrane, the electrostatic interaction between the charged groups, and the hydration energy of the polar head groups manifested as the hydration pressure.^19−22^ For neutral molecules, the thermal undulation and hydration energy will contribute to the repulsive force. The balance between attractive and repulsive forces determines the formation of stable single-layer or multilayer liposomes with an equilibrium lamellar spacing, *d*, and ultimately determines the formation of unilamellar or multilayer versicles.^21,23^ Therefore, a change in *d* indicates a shift in the balance between the attractive and repulsive forces. The presence of salt can modify both the attractive and repulsive forces at low salt content; however, the electrostatic interaction should be screened at sufficiently high salt concentration. Kučerka et al. found that different mechanisms of interaction contributed to ion-bilayer interaction.^24^ At small interlipid distances the lipids formed lipid-ion-lipid bridges which in turn lead to a thickening of the bilayer due to a more ordered assembly of the hydrocarbon tails.^24^ At larger interlipid distances, a single ion paired with a single lipid headgroup.^24^

Here, we present experimental observations of previously unknown discontinuities in the structural parameters, like liposome diameter and membrane thickness. We explain that such concentration-dependent effects arise from structural transitions due to the formation of additional bilayers caused by increasing the osmotic pressure and reducing the solvent quality. Pinpointing the exact osmotic pressures at which the increase in the number of bilayers occurs allows for precise tuning of vesicle lamellarity and thus stability.

## Methods

### Materials

Highly purified (>99%) 1,2-di(octadecenoyl)-*sn*-glycero-3-phosphocholine (DOPC) was purchased from Avanti Polar Lipids (Alabaster, AL, USA) and used without further purification. Biotechnology grade sodium chloride (NaCl) (99.9% purity) was obtained from VWR Life Sciences (Solon, OH, USA), organic solvents (HPLC grade), and D_2_O were purchased from Sigma-Aldrich (St. Louis, MO, USA).

### Sample Preparation

DOPC liposomes were prepared by dissolving DOPC lipid powder in chloroform, removing the chloroform using a rotary evaporator, and drying under a vacuum overnight. The dried lipid was hydrated using D_2_O, and the resultant solution was subjected to freeze–thaw cycling by alternatingly immersing the flask in water at around 50 °C and placing it in a freezer at −20 °C for 10 cycles in 10 min intervals. Finally, the solution was extruded using a mini extruder (Avanti Polar Lipids, Alabaster, AL, USA) through a polycarbonate membrane with a pore diameter of 100 nm (33 passes) to obtain unilamellar liposomes. Liposome solutions prepared in D_2_O were mixed with NaCl solutions in a 1:1 ratio to achieve the desired extravehicular NaCl concentration, and a 24 h waiting time ensured that samples were in their best equilibrium states. Unless stated otherwise, all experiments were conducted at ambient temperature (23 °C) and DOPC concentration of 0.25 wt %.

### Dynamic Light Scattering (DLS)

DLS measurements were performed using a Malvern Zetasizer Nano ZS equipped with a He–Ne laser of wavelength, λ = 633 nm at 30 mW laser power, at a scattering angle θ = 173°. The hydrodynamic radius, *R*_h_, of the liposomes in each NaCl concentration was calculated using the Stokes–Einstein equation, *R*_h_ = *k*_B_*T*/(6πη_0_*D*), with the Boltzmann constant, *k*_B_, the temperature, *T*, the viscosity of the solvent (D_2_O or NaCl solution), η_0_. Four separate DLS measurements for each mixture were averaged.

### Zeta Potential

Zeta-potential measurements were done using the Next Generation Electrophoretic Light Scattering (NG-ELS) instrument with extended capabilities to allow measurements at high salt concentrations. Approximately 0.5 mL of each sample was placed in a disposable 4 mm path-length cuvette with blackened Platinum electrodes, significantly reducing electrode polarization. Five measurements of each sample were carried out for 60 s at 8 or 10 V at 64 or 128 Hz.

### Cryo-Transmission Electron Microscopy (Cryo-TEM)

Cryogenic transmission electron microscopy (cryo-TEM) images were recorded on a Tecnai G2 F30 operated at 150 kV. A volume of ten microliters of the sample (0.125 wt % DOPC: in pure D_2_O, or NaCl) was applied to a 200-mesh lacey carbon grid mounted on the FEI Vitrobot plunging station, and excess liquid was blotted for 2 s by the filter paper attached to the arms of the plunging device. The carbon grids with the attached thin film of liposome suspensions were plunged into liquid ethane and transferred to a single-tilt cryo-specimen holder for imaging. By quickly plunging into liquid ethane, the vesicles are preserved at their hydrated state at room temperature. Cryo-TEM images were obtained in the bright field setting.

### Small-Angle X-ray Scattering (SAXS)

SAXS experiments were conducted at the LIX beamline at the National Synchrotron Light Source II, Brookhaven National Laboratory, and at the Bio-SAXS beamline at the Stanford Linear Accelerator Center (SLAC) facility. The samples were measured in a flow cell with an acquisition time of 1 s at the synchrotron instrument. The samples were loaded in 1 mm borosilicate glass capillary cylinders for the lab X-ray with an acquisition time of 10 s. The recorded intensities were corrected for dark current, empty cell, and solvent (buffer) using standard procedures.^25,26^ The scattering intensity was normalized to absolute units (cm^–1^) using water as the calibration standard.^27^ The data modeling is explained in the Supporting Information (SI).

### Small-Angle Neutron Scattering (SANS)

SANS experiments were conducted at the NG 7 SANS instrument of the NIST Center for Neutron Research (NCNR) at the National Institute of Standards and Technology (NIST).^28^ The sample-to-detector distances, *d*, were fixed to 1, 4, and 13 m, at neutron wavelength, λ = 6 Å. Another configuration with lenses at *d* = 15.3 m, and λ = 8 Å was used to access low *Q*s.^29^ This combination covers a *Q*—range from ≈0.001 to ≈0.6 Å^–1^, where *Q* = 4π sin (θ/2)/λ*,* with the scattering angle, θ. A wavelength resolution of, Δλ/λ = 14%, was used. All data reduction into intensity, *I*(*Q*)*,* vs momentum transfer, *Q* = |*Q⃗*|, was carried out following the standard procedures implemented in the NCNR macros for the Igor software package.^30^ The intensity values were scaled into absolute units (cm^–1^) using a direct beam. The solvents and the empty cell were measured separately as backgrounds. The data modeling is presented in the Supporting Information (SI).

## Results and Discussion

Hereafter, we report on changes in the structural parameters observed after adding salt to liposomes in an aqueous solution. Since the NaCl has been added after the self-assembly of liposomes, the inner compartment is free of salt, at least at the beginning. We test how the vesicular system responds to this initial imbalance.

### Vesicle Size and Surface Charge

**Figure 1:**
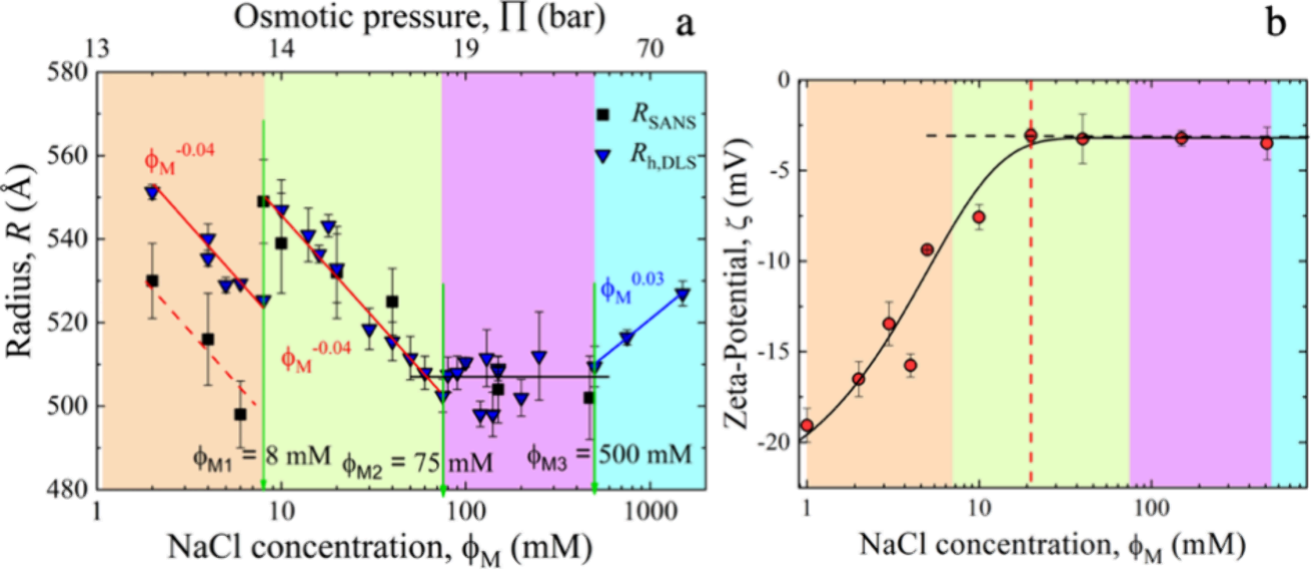
(a) Vesicle radius in lin-log representation from dynamic light scattering (DLS) with increasing NaCl concentration, ϕ_M_. The vertical arrows indicate the transition concentrations at 8, 75, and 500 mM. SANS results show the same trend. (b) Zeta-potential data as a function of ϕ_M_. The solid line represents continuous growth. In panels (a) and (b), the four different regions are distinguished by different colors.

Increasing the ion concentration of solutions of liposomes and water leads to higher osmotic pressures, and a continuous reduction of the vesicle diameter is the logical consequence. However, Figure 1a illustrates that at least four different regions can be distinguished. (1) An initial sharp size reduction with a power-law, ϕ_M_^–0.04 ± 0.003^, as observed from independent DLS and SANS experiments. (2) There is an abrupt increase in size at ϕ_M1_ = 8 mM but it continues to decay with same the power law, ϕ_M_^–0.04 ± 0.003^. (3) At higher concentrations from 75 to 500 mM, we observe a constant size within experimental accuracy. (4) At concentrations above, ϕ_*M*3_, we find a slight increase in size with a weak power-law dependence, ϕ_M_^0.030 ± 0.001^. The osmotic pressure axis was calculated using results presented by Luo et al.^31^ The conversion can be seen in Figure SM4 of the SI.

It is well-established to assume that salt perturbs the equilibrium between electrostatic repulsion and van der Waals attraction between the lipids. Measuring the zeta potential (or ζ-potential) provides more information about the charge on the vesicle surface. Figure 1b indicates a continuous increase of the zeta potential with the NaCl concentration. Assuming an exponential increase can describe the data and provide a growth constant of 6 ± 1 mM until it reaches its plateau at around 20 mM. This 20 mM indicates a characteristic change of the zeta potential, but none of the three discontinuities, at 8, 75, and 500 mM, seems to be connected. Hence, the zeta potential change also appears unlikely to be related to the observed discontinuities.

As both osmotic pressure and electrostatic interaction alone are unlikely to be the sole reason, we can ask whether the balance between the osmotic pressure and Coulomb interactions may be responsible for the effects observed at 8, 75, and 500 mM. To clarify this question we investigate the structure of the vesicle as a function of the concentration in more detail, using the additional techniques, cryo-transmission electron microscopy (cryo-TEM), SAXS, and SANS.

### Vesicle Morphology Revealed by Cryo-TEM

**Figure 2:**
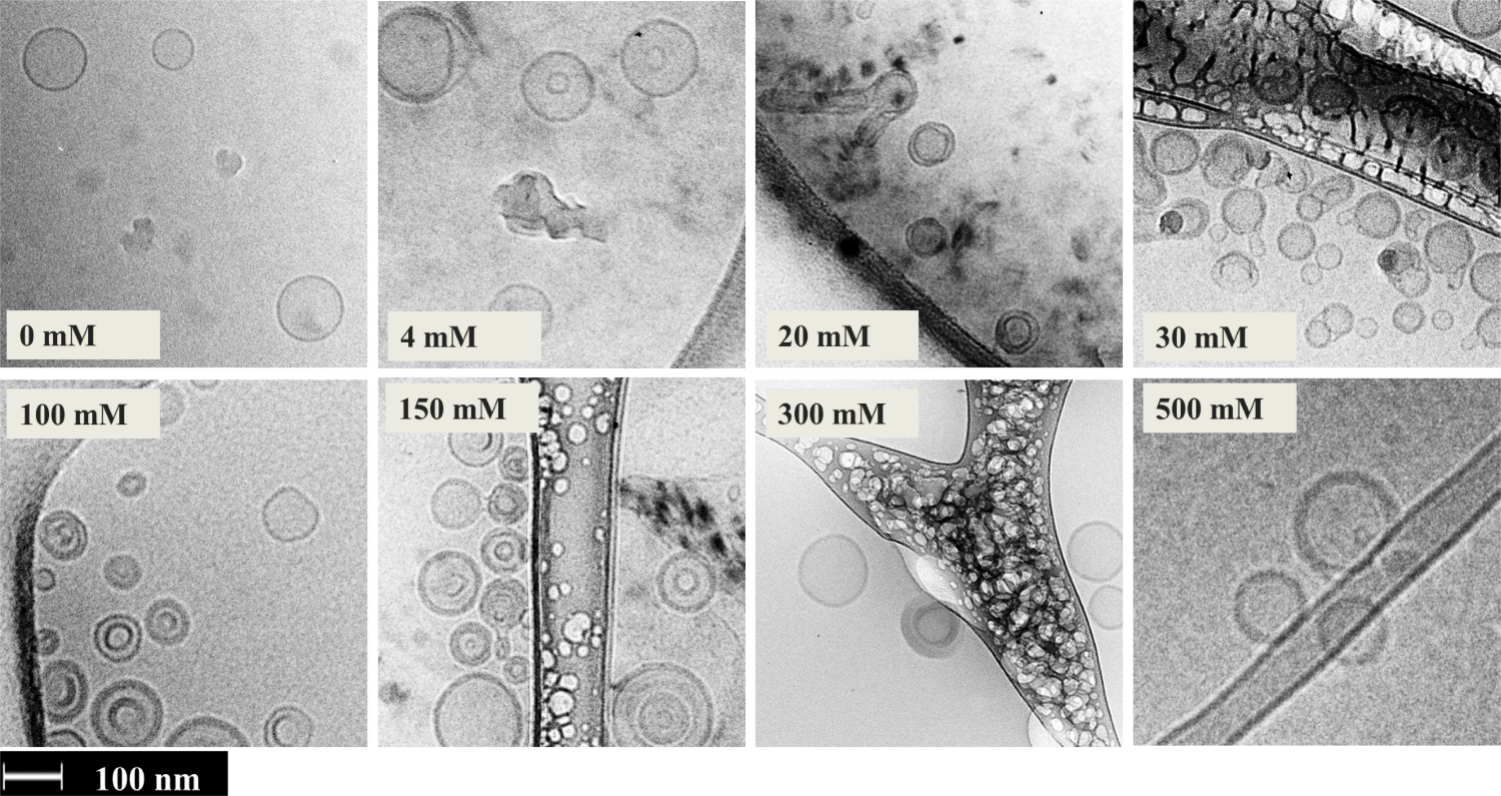
Cryo-TEM (cryogenic transmission electron microscopy) images of DOPC liposomes in D_2_O exposed to different NaCl concentrations, as indicated in the photos. The horizontal bar on the bottom represents 100 nm.

Cryo-TEM images in Figure 2 indicate a reduction of the diameter caused by the presence of salt, which is accompanied by a broadening of the circular boundary layer. This boundary layer broadening is likely related to a transition from unilamellar to multilamellar liposomes. Noteworthy, the transformation into bilamellar vesicles occurs already at shallow salt content of, ϕ_M_ < 20 mM, comparable with human cytosolic salt concentration. At higher salt content ϕ_M_ ≥ 20 mM, cryo-TEM images reflect a change of the liposome diameter and formation of double or triple-layered multilamellar vesicles (MLVs) along with increased polydispersity. The cryo-TEM image, for ϕ_M_ ≥ 100 mM, shows a mixture of fused and unilamellar vesicles, indicating a change to a heterogeneous system.

While the cryo-TEM experiments already indicate the influence of salt on the structure, including the diameter and the number of bilayers, scattering techniques can add more information on the statistical significance. Hence, conducting scattering experiments is relevant to show whether enough liposomes show the transition observed by cryo-TEM to be connected to the discontinuities, as illustrated by Figure 1a. Hereafter, we present both SANS and SAXS. While SANS provides essential information on the size and shape of liposomes, the scattering length and associated contrast by the phosphorus head groups for X-rays make SAXS the perfect tool to explore changes in the bilayer, including thickness and number of lamellae.

### Vesicle Structure from SANS

**Figure 3:**
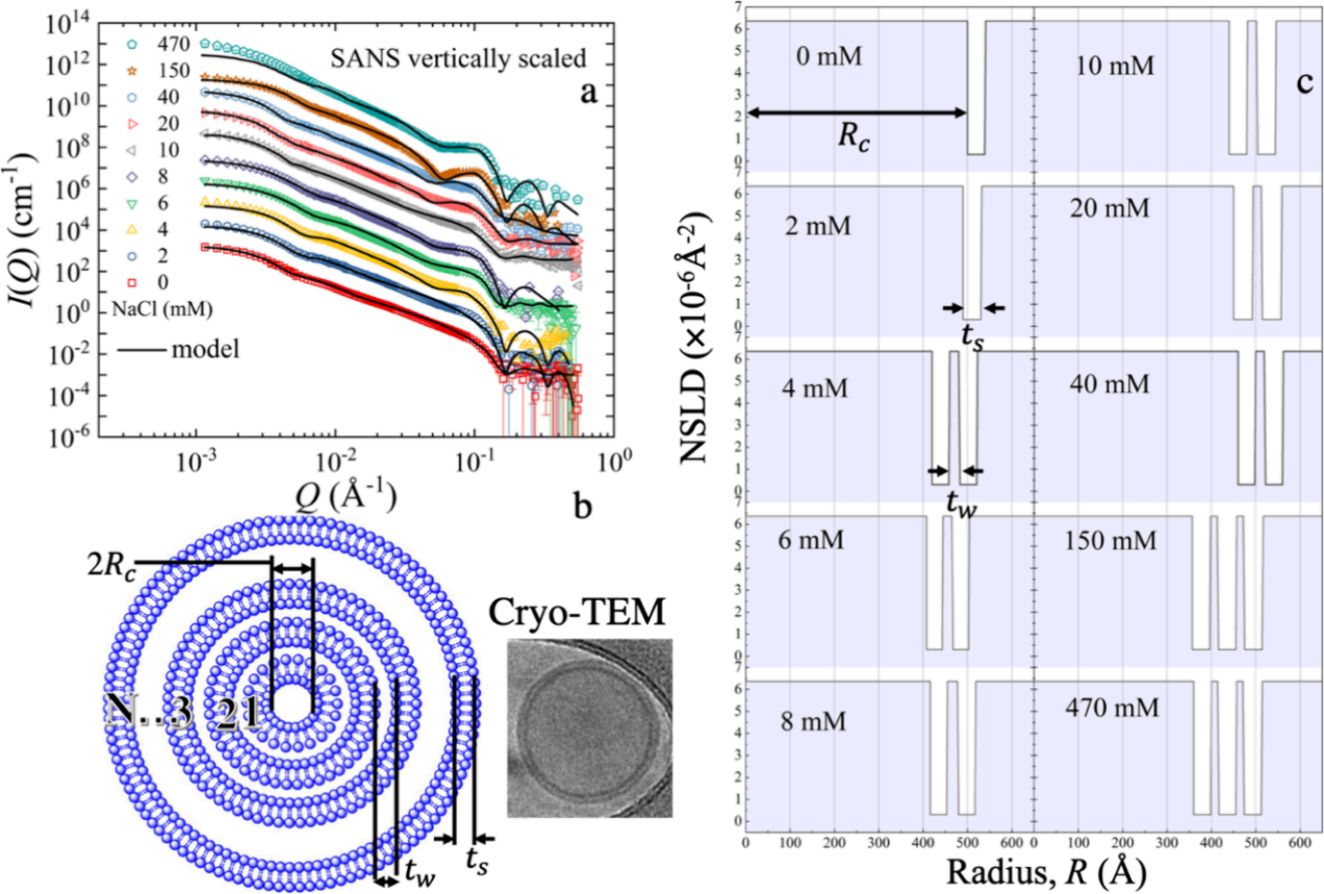
(a) SANS scattering intensity for DOPC in D_2_O and different salt (NaCl) concentrations, ranging from 0 to 470 mM. The solid line represents the fits using the form factor for a unilamellar vesicle for 0 and 2 mM and a multilamellar vesicle for 4–470 mM samples. The data are vertically scaled for better visualization by multiplication with a constant value in a logarithmic scale. (b) Schematic representation of the multilamellar liposome illustrating the number of bilayers, *N*, the diameter of the core, 2*R*_c_, the thickness of the individual shells, *t*_s_, the thickness of the interleaved solvent layers, *t*_w_. Cryo-TEM image for the formation of MLVs. (c) Neutron scattering length density (NSLD) profile as a function of vesicle radius for different salt concentrations. The data modeling is presented in the Supporting Information (SI).

The SANS results for samples prepared with different salt concentrations are plotted in Figure 3a. The intensity is scaled vertically for better visualization. Scattering diagrams represent the statistical average of the morphology of liposomes, including the diameter and number of lamellar layers. The intensity vs momentum transfer, *Q*, plots of the different concentrations decay from higher to lower intensity with a characteristic decay that allows us to determine the structure. Here, we used a model represented by the (black) full lines. The most apparent differences involve the growing peak intensity at higher *Q*s, indicating a multilamellar structure’s emergence. Even at concentrations as low as 4 mM, the first signs of a statistical amount of multilamellar structures are visible. This observation is notable because 4 mM is well in the region of the human cytosolic salt concentration (4–12 mM).

Mathematical modeling provided a more detailed analysis of the changes in vesicle radius with salt concentration. The solid lines in Figure 3a represent a multilayer vesicle form factor, with details summarized in the Data Modeling section of the SI. The model assumes a water compartment (core) of radius, *R*_c_, surrounded by *N* lipid bilayers, each of thickness, *t*_s_, separated by *N*-1 inter water layers, each of thickness *t*_w_.^32^ The number of layers and changes in the distances from the center are illustrated by the corresponding neutron scattering length density profile in Figure 3c.

The best description of the SANS data in Figure 3a can be obtained by including a Gaussian distribution for the interbilayer water and lipid bilayer thickness. Numerical values from the fits are compiled in Table S1 of the SI. The width of the distribution was kept less than 0.1 for *t*_s_, though a broader thickness distribution for *t*_w_ ranging from 0.6 at low to 0.8 at a higher salt concentration (40 mM) is necessary. The interbilayer water thickness, *t*_w_, is practically unchanged (∼20 Å) for lower salt concentrations ≤40 mM; however, a sharp reduction of *t*_w_ to ∼15 Å occurs at 150 and 470 mM.^33^

Hence, the SANS results confirm that a statistical number of liposomes changes the radius and transitions from unilamellar to MLVs. The number of lamellae increases with the increase in concentration. However, there are three concentration regions, with 1, 2, and 3 bilayers. Before we connect multilayer formation to the size changes observed by DLS in Figure 1a, the changes in the lamellar layers are studied in more detail using SAXS.

### Membrane Structure from SAXS

While SANS provides information on the liposome diameter, SAXS further refines our knowledge of the thickness and number of layers. Compared to neutrons, the X-ray contrast for the lipid heads with the phosphorus atoms is higher, and the instrumental resolution of X-rays is better. Hence, from a theoretical point of view, SAXS information on the bilayer structure should be more accurate.

**Figure 4:**
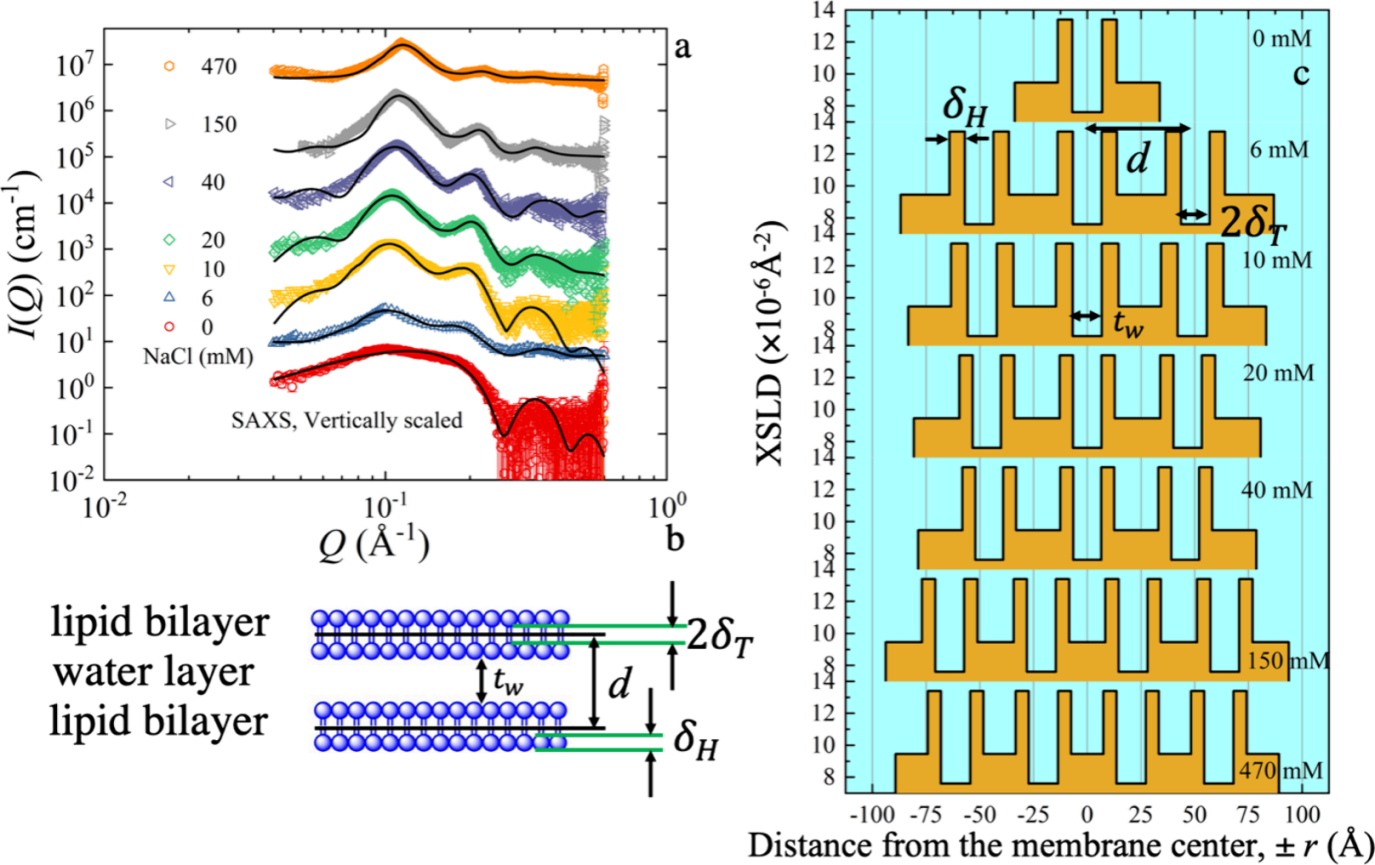
(a) SAXS scattering intensity for DOPC in D_2_O with different salt (NaCl) concentrations, ranging from 0 to 470 mM. The solid line represents the fits using a lamellar structure factor. The data are vertically scaled for better visualization by multiplication with a constant value in a logarithmic scale. (b) Schematic representation of the lipid multilamellar structure illustrating the thickness of the lipid head, δ_H_, the thickness of the lipid tail region, δ_T_, the water layer thickness, *t*_w_, and the lamellar repeat distance, *d*, of bilayers. (c) X-ray scattering length density (XSLD) profile as a function of distance from the membrane center for different salt concentrations. The data modeling is presented in the Supporting Information (SI).

SAXS results are presented in Figure 4. For a better comparison, the scattering intensity is vertically scaled. In the absence of salt, the first-order diffraction peak yields a repeat distance,  63 ± 1 Å. This value is close to 63.1 ± 0.3 Å, previously reported for oriented stacks of unilamellar vesicles.^34^ Similar to the SANS data in Figure 3, we observe a peak that grows with increasing salt concentration. Hence, the SAXS and SANS data appear to be compatible.

The Caille structure factor, with details presented in the Data Modeling section of the SI, was used to find more information on the lamellar sheets sketched in Figure 4b.^35,36^ The results permit direct access to the lamellarity or the number of repetitive multilayers, *N*, the lamellar repeat distance, *d*, as well as the thickness of the lipid head and tail groups, represented by δ_H_ and δ_T_, respectively. The head-to-head bilayer thickness is given by δ_HH_ = 2(δ_H_ + δ_T_). The position of the first-order Bragg peak is given by *Q*_0_, *k*_B_ is Boltzmann’s constant, and *T* is the absolute temperature. More details of the model can be found in the Data Modeling section of the SI.

Figure 4c shows the results of the numerical fits for different concentrations. Numerical values from the fits are compiled in Table S2 of the SI. The increase in the number of layers and changes in the distances to liposome centers with the ion concentration is illustrated by the X-ray scattering length density profile shown in Figure 4c. Additionally, we calculated the ratio of the first, second, and third peak positions, *Q*_1_:*Q*_2_:*Q*_3_ = 1:2:3 (Figure 4a). This calculated ratio independently confirms lamellarity with well-defined repetitive distances.^37^ Formation of higher-ordered lamellar structures (larger *N*) is further confirmed by a much sharper first-order diffraction peak, corresponding to more regular lamellar spacing than at low salt concentrations. The best model description of the data was accomplished with *N* = 3 ± 1 layers, for the lower NaCl concentrations, and *N* = 4 ± 1 layers for salt concentrations of 150 and 470 mM, respectively. The better contrast and higher resolution of SAXS enabled more layers to be resolved than in the SANS, *N* = 2 (up to 40 mM), and *N* = 3 ± 1 (150 and 470 mM). The values agree with the statistical accuracy.

**Figure 5:**
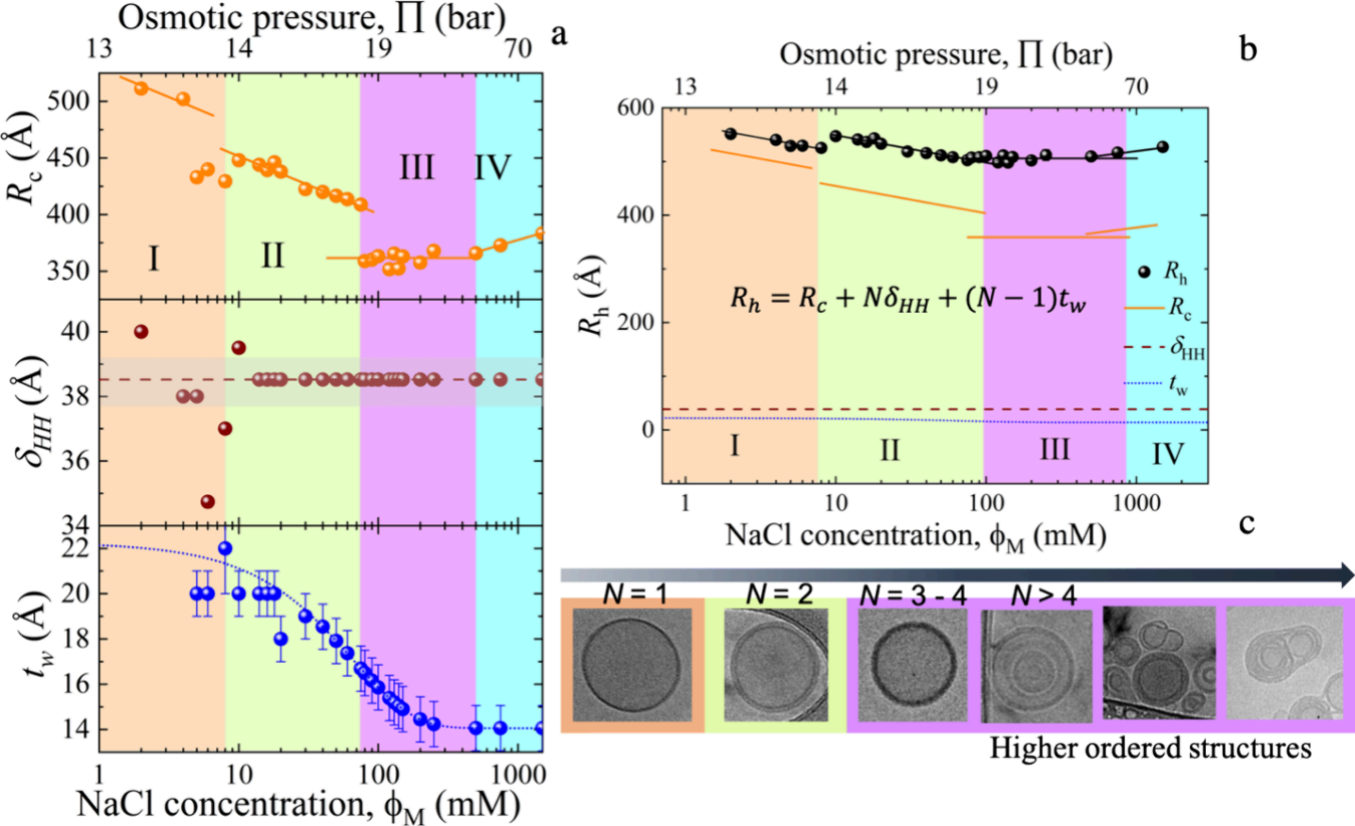
The different color codes illustrate four distinct phases with increased salt concentration. (a) Core radius of the vesicle, *R*_c_, the bilayer thickness, δ_HH_ = 2(δ_H_ + δ_T_), and the distance between the bilayers as given by the water layer thickness, *t*_w_, are plotted as a function of the salt concentration, ϕ_M_, (bottom axis) and the osmotic pressure, Π (top axis). *R*_c_ follows the same power-law dependence as *R*_h_ in Figure 1, represented by the solid lines. The average δ_HH_ is given by the horizontal line and its standard deviation is indicated by the shaded area. The *t*_w_ exhibits a logarithmic concentration dependence that can be described by a theoretical model that relates osmotic pressure and *t*_w_ by, Π= *P*_0_exp (*−t*_w_/λ), for an applied pressure, *P*_0_, over a decay length.^20^ (b) *R*_h_ from Figure 1 is replotted to compare it with *R*_h_*= R*_c_+ *N*δ_HH_ + (*N –* 1)*t*_w_. (c) Cryo-TEM images show the evolution from ULVs to MLVs and different higher-order structures with increased salt concentration (arrow).

In the next step, the results are analyzed to determine whether these structural changes can explain the size decrease with the increasing salt concentration and the discontinuities at specific concentrations. Modeling parameters from SANS and SAXS are compiled in Figure 5a. While a virtually constant δ_HH_ was observed, the thickness of the water layer, *t*_w_, initially decreased from 22 to 14 Å but stayed virtually constant at high concentrations (>250 mM). The one-order of magnitude more substantial decrease of about 150 Å of the core size contributes more strongly to the entire liposome diameter, as visualized by Figure 5b. As core and liposome diameters decreased with increasing salt concentration, the discontinuities have a different origin.

As illustrated by the black line in Figure 5b, the hydrodynamic radius of the liposome was calculated from *R*_h_ = *R*_c_ + *N*δ_HH_ + (*N* – 1)*t*_w_. This expresses the importance of the formation of multilayers for the size discontinuities. Independent observations of the formation of these multilayers by cryo-TEM are illustrated by the images in Figure 5c. The formation of multilayers explains the apparently contradicting observation of the simultaneous expanding liposome and shrinking core diameter at 8 mM, which is also the reason for the diameter decrease with increasing concentration and the sudden increase at 8 mM.

The water layer thickness, *t*_w_, decreased with increasing salt concentration. A theoretical description can be based on the observations for zwitterionic lipid bilayers that below the equilibrium bilayer separation, the interbilayer repulsive force falls of exponentially over a decay length, λ, which leads to Π = *P*_0_ exp (−*t*_w_/λ), with net repulsive pressure, *P*_0._(20,38,39) The corresponding pressure distance plot that shows fitted data is presented in Figure SM5, SI. A decay length, λ, of around 1 Å was determined by fitting the data. Interbilayer water thickness values in the 14–20 Å range correspond to *P*_0_ = (4.9 ± 1.0) × 10^7^ bar. From this value, Π = 40 bar at equilibrium has been calculated using an interbilayer distance of 14 Å, at a salt concentration of 470 mM. The osmotic pressure is 39 times higher than the normal atmospheric pressure. Therefore, at high salt concentrations, Figure 5b reflects the diverging nature of the repulsive force, preventing individual bilayers from coming in close contact. To maintain a balance with the osmotic force, the interbilayer repulsive force increases exponentially, exp (−*t*_w_/λ), accompanied by the formation of higher-order MLVs. The influence of the osmotic pressure on the bilayer thickness, δ_HH_, is negligible, cf. Figure SM4, SI. Thus, the lamellar repeat distance, *d* = *t*_w_ + δ_HH_, follows the concentration dependence of *t*_w_. The influence of *t*_w_ on the liposome diameter, *d*, is less important because of *t*_w_ ≪ *R*_c_.

Hence, the experiments provide a plausible explanation for the observed size reduction and discontinuities in DOPC with increasing salt concentration.

The increase of the liposome diameter and the decrease of the core size is well compatible with the emergence of new bilayers, with the remarkable result that individual layer thickness stays virtually constant. The thickness of the interbilayer water shows a continuous concentration dependence instead of abrupt changes observed in the liposome radius. Hence, we conclude that the observed continuous decrease of the diameter by DLS is a consequence of the change of the interbilayer, while the formation of new bilayers causes discontinuities.

With this correlation between diameter and molecular structural parameters, the molecular parameters are explored in more detail in the next step. The first observation of a continuous change in the thickness of the interbilayer water is already a fundamental key observation, which requires a pressure difference between interbilayer and bulk water. As the experiments showed the formation of MLVs, ions could intrude into the interbilayer water during this reassembly. However, the thickness decrease requires a positive pressure, hence a lower salt concentration inside than outside.

To answer the question of ions in the interbilayer water in more detail, the dependence of the diameter decreases on the concentration below and above 8 mM, which can be compared. The experimentally observed exponents of the power laws that describe the diameter reduction of the core below and above 8 mM are the same within the experimental accuracy. Initially, there is no salt inside the membrane, and there is no reason that salt intrudes into the intact membrane below 8 mM. Since the exponent is the same for the ranges <8 mM and 8 mM < ϕ_M_ < 75 mM, the repulsive force seems unchanged in region II. Hence, the observation of the same exponent implies that the concentration difference below and above 8 mM is the same, which leads to a concentration of salt equal to zero in the core.

Within region two, an exponent for the interbilayer water layer follows a power law Φ_M_^–0.15^. This is 1 order of magnitude steeper than the core or liposome. Given the limited concentration window, we want to interpret this value sparingly. However, because this change is at least of comparable order of magnitude with the core shrinkage, the salt or salt enrichment in the interbilayer water layer for ϕ_M_ < 75 mM can be excluded.

The constant thickness of the interbilayer water is a consequence of salt intrusion, which causes the cryo-TEM observation of mixtures of unilamellar and fused vesicles at ϕ_M_ ≥ 100 mM (regions III and IV in Figure 5). The salt concentration in the water inside and outside the vesicles is the same in regions III and IV.

**Figure 6:**
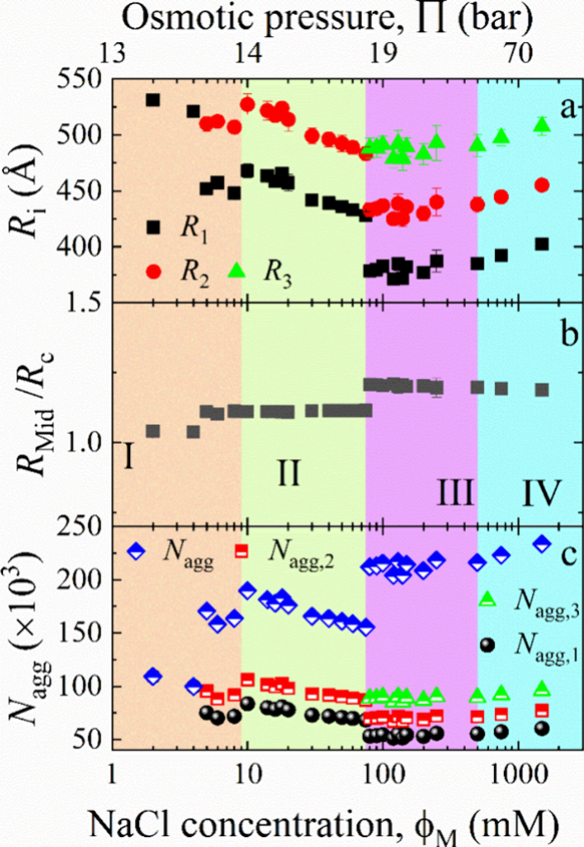
(a) Distances *R*_1_, *R*_2_, and *R*_3_ of the bilayer midplanes for bilayer 1, bilayer 2, and bilayer 3, respectively, from the center of the liposome, or short *R*_Mid_ ≔ *R*_Mid,*N*_ with *N* = 1, 2, 3. (b) Distance of the bilayer midplane from the vesicle center, *R*_Mid_ = *R*_c_*+ N*δ_HH_/2 + (*N –* 1)*t*_w_/2, as a function of salt concentration, ϕ_M_. (c) Aggregation number, *N*_agg_, given by the number of lipid molecules per vesicles is presented as a function of ϕ_M_, and the osmotic pressure, Π. A comparison has been made to the number of lipids in the inner, middle, and outer shells of a MLV given by *N*_agg*,*1_, *N*_agg*,*2_, and *N*_agg*,*3_, respectively. Here, *N*_agg_ = *N*_agg*,*1_+ *N*_agg*,*2_ + *N*_agg*,*3_.

The results presented in Figure 5 establish the formation of multilayers as the origin of the size discontinuities. In the next step, the individual bilayers are examined. For more straightforward wording, the numbers 1, 2, and 3 indicate the inner to the outermost bilayer. The changes in the distances of the bilayers, *R*_1_, *R*_2_, and *R*_3_, from the center of the liposomes as a function of the salt concentration are illustrated in Figure 6a.

Only one bilayer exists below 8 mM (region I) with the radius *R*_1_. In the range of 8–75 mM (region II), a second layer emerges. The radius of the second layer *R*_2_ is greater than the radius of *R*_1_ in the region I (<8 mM). However, *R*_1_ in region 2 is lesser than *R*_1_ in region 1. This already mirrors that the existence of layer 2 is responsible for the size discontinuity observed by DLS (Figure 1). Inspecting region III from around 75 to 500 mM reveals a change in the concentration dependence. However, continuous transitions from layer 2 to layer 3 and from layer 1 to layer 2 are observed. These results suggest that adding salt creates one layer closer to the center, but the diameter of the others stays essentially the same. Such behavior is not observed transitioning from Region III to Region IV, where *R*_1_, *R*_2_, and *R*_3_ do not show any visible discontinuity. Instead of a size decrease a diameter increase of each bilayer is observed. Another observation is the similar concentration dependence of the different radii visible in Figure 6a.

The discontinuity is also visible in the change of the distance of the bilayer midplane from the vesicle center, *R*_Mid_ = *R*_c_ + *N*δ_HH_/2 + (*N* – 1)*t*_w_/2, which abruptly changes at the transition concentrations, but stays constant within the regions.

More details can be revealed by calculating the number of lipids in each bilayer, *N*_agg_, also called the aggregation number. The numbers follow the same convention: 1, 2, and 3 from the inner to the outermost layer, respectively. The total number of lipids in each liposome can be calculated as the sum of the lipids in each layer, e.g., *N*_agg_ = *N*_agg,1_ + *N*_agg,2_ + *N*_agg,3_.

As illustrated in Figure 6c, numbers, *N*_agg_, show a concentration dependence and discontinuities. The innermost layer has the minimum number of lipids, and the outermost layer has the most lipids. We also notice that the number of lipids per liposome increased with increasing the concentration, except for region II. However, the decrease in region II is less than the increase during the transition from region I to II and from II to III.

**Figure 7:**
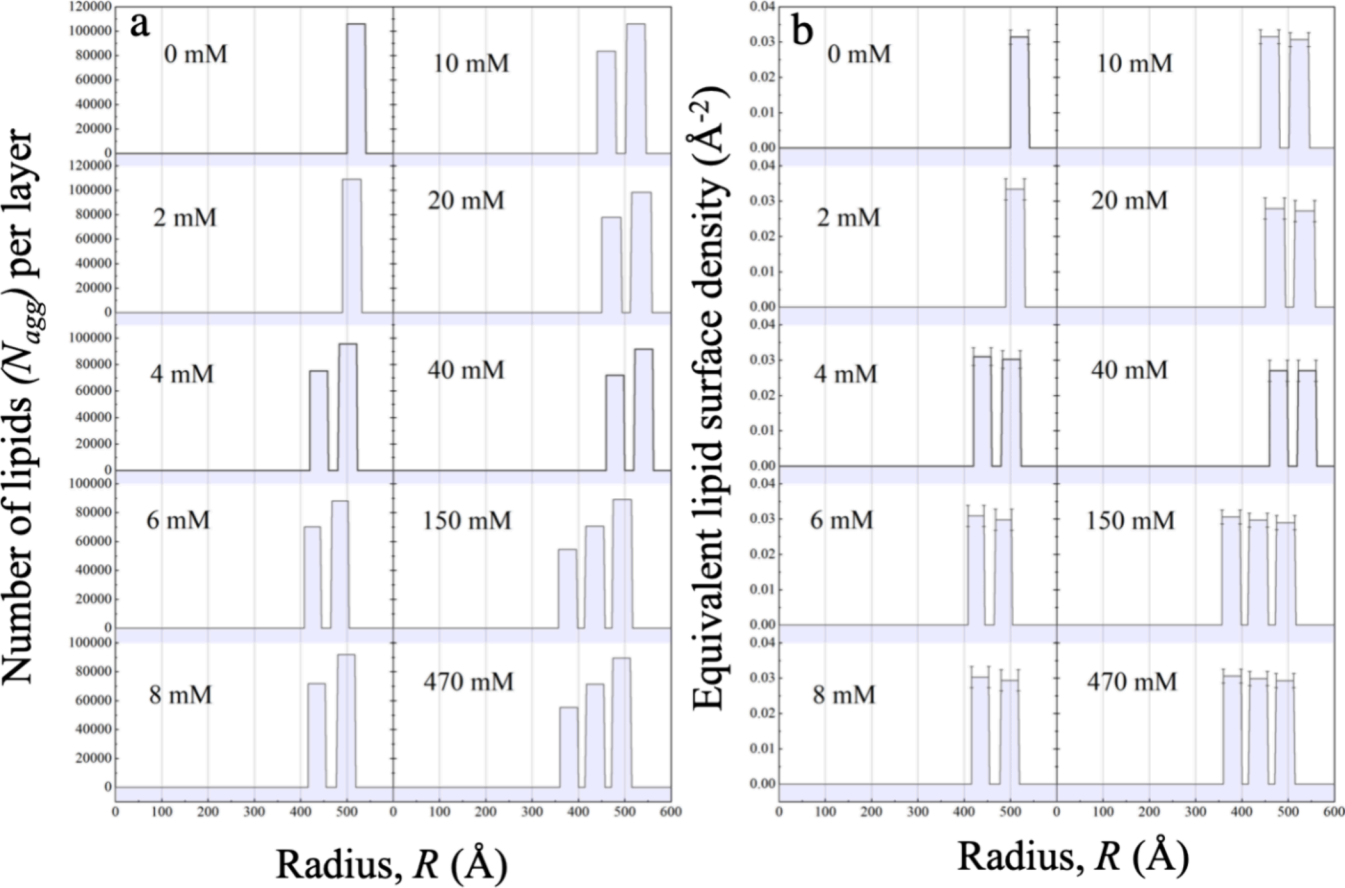
(a) Number of lipids per layer, *N*_agg_, as a function of the radius or the distance from the center of the vesicles. (b) Equivalent lipid surface density on each layer as a function of the radius. For each layer it is calculated on the surface of the bilayer midplane and represents a uniform surface density, 0.030 ± 0.003 Å^–2^.

Given the observation of a size change with the concentration, the changing number of lipids could result from the geometrical packing of the lipids in the layer. Hence, Figure 7a displays the number of lipids, and Figure 7b the equivalent lipid surface density, both as a function of the distance from the liposome center.

While Figure 7a shows a change in the number of lipids in each layer, the equivalent lipid surface density stays constant within the experimental accuracy, *N*_agg_/(4π*R*_*i*_^2^) = 0.030 ± 0.003 Å^–2^. Hence, it is likely that an additional layer is created to maintain the equivalent lipid surface density. Since all lipid heads occupy approximately the same space for both the inner and outer leaflets of each bilayer, the inner layer starts to relieve the pressure by releasing lipids first.^40^ This explanation is also compatible with the transitions from region I to II and from region II to III, leading to a layer that is continuous.

The elastic energy of liposomes is proportional to the relative change in the surface area. If the compression becomes too large, one bilayer may no longer be able to accommodate all lipids. Thus, a discontinuity may arise from a spontaneous relief by releasing lipids from the bilayer. This is supported by the fact that the lipids prefer maintaining a constant surface density in each layer. It is given by *N*_agg_/(4π*R*_*i*_^2^) = 0.030 ± 0.003 Å^–2^. This is presented in Figure 7b. In our case, the change in the distance between the bilayers is not enough to see an influence of curvature on lipid density. The experimental data shows the emergence of new bilayers with smaller and larger diameters compared to the results for 0 mM concentration. Since the released lipids lead to an increase in the concentration of free lipids in the system, we expect these may form new bilayers. As obtained from DLS in Figure 1a, the size was reduced by 9%, whereas the surface area of the vesicles was reduced by 18% at 75 mM. At high salt content from 75 to 500 mM, the size and the surface area appear to be virtually constant, followed by a 3% increase in size and a 7% increase in surface area between 500 and 1500 mM. When the number of lipids per vesicle is approximately 2 times more than in ULVs, we observe formations of MLVs. The shrinkage in size and the formation of MLVs are intertwined. The balance between the outside osmotic pressure and the interlayer repulsive force of the membranes can control the change in the size of the vesicles. The corresponding repulsive pressure increases dramatically to 39 atm for a salt concentration of 470 mM. However, as the SANS/SAXS results illustrate, internal layers are created even at these high concentrations.

Let us first consider one bilayer to expand this discussion toward a quantitative understanding. For DOPC it was observed that the inner and outer monolayers are identical with slight dependence on the vesicle curvature.^40^ From X-ray diffraction results shown in Figure 4a, for a thickness, δ_HH_ = 41 Å, at 0 mM salt concentration, the number of lipids per vesicle in one shell for ULVs is given by, *N*_agg,1_ = *V*_s_/*V*_l_ = 1 × 10^5^, for a vesicle of *R*_h_ = 551 Å (DLS), with *V*_s_ being the outer shell volume of the ULV and *V*_l_ the molar volume of the phospholipid. With an increase in salt content to 150 mM the size of the ULV of thickness, δ_HH_ ≈ 39 Å (SAXS), shrinks to *R*_h_ = 509 Å, and will have the number of lipids per vesicle, 9 × 10^4^. Therefore, under this assumption of the formation of ULVs, there will be an excess of 2 × 10^4^ lipid molecules per vesicle in the solution. The excess lipid concentration is equivalent to a monolayer membrane surface, that can be accommodated into MLVs. As explained below, the increasing zeta potential indicates a lower solubility of the outermost layer, further pointing to generating new layers inside the internal compartment. Figure 6c illustrates this discussion by plotting the number of lipids per vesicle aggregation number, *N*_agg_, with salt concentration. This shows an excess number of lipids per vesicle determined by *N*_agg_ at each transition concentration. We have *N*_agg_ by a factor of 2 higher than in the absence of salt to observe the transition to MLVs.

In the next step, we need to understand the continuous size reductions in regions I and II underlying the discontinuity at 8 mM. If we compare the effect of salt on vesicle size above and below the transition concentration ϕ_M1_ = 8 mM in Figure 1a, we observe a power-law decay of the vesicle size for ϕ_M_ ≤ 80 mM is observed with the same power law exponent in the entire region.

The number of layers, *N*, in our MLVs, is almost constant in region two of the semidilute salt concentrations, ϕ_M1_ < ϕ_M_ < ϕ_M2_. In addition to the existence of weak attractive van der Waals force between the lipid layers which only dominates at very low salt concentrations, another possibility is hydration attraction or H-bonding across a water layer of thickness *t*_w_ due to complementary surface polar head groups.^41^ Rand et al. theoretically predicted such a mechanism in understanding the membrane’s surface hydration.^41^ In this case, partial dehydration of the lipid heads should further facilitate hydration attraction at high salt concentrations.

At low salt concentrations, the zeta-potential data also show a net decrease of the negative charge on the vesicle surface which reaches a plateau at 20 Mm (Figure 1b). The growth constant of 6 ± 1 mM is close to the first transition concentration, ϕ_M1_ = 8 mM. The sign and the magnitude of the zeta potential are determined by the net charge deposited on the vehicle’s surface. Phospholipid headgroups carry a negative charge (phosphate group) and a positive charge (choline group), resulting in a zwitterionic nature (no net charge) at neutral pH. Despite their zwitterionic nature, the observed effect of a weak negative potential is prevalent for phospholipids. Molecular dynamics simulations point to water molecules at the surface of the liposomes directed toward the negatively charged phosphate groups. This preferred orientation generates a layer of positive charge near the surface, hence the observance of a negative zeta potential.^42^ The polar head groups are known to reorient with increasing ionic strength.^43^ This phenomenon is further facilitated by the osmotic pressure. Therefore, Na^+^ ions bind to the phosphate groups of the lipid head, and Cl^–^ ions bind to the trimethylammonium, N^+^(CH_3_)_3_. This causes an increase in surface charge density at the interface of water and the polar lipid headgroup (Figure 1c). The increasing zeta potential might cause localized surface insolubility which further contributes to the formation of MLVs. A comparison of the vesicle size and zeta potential from Figure 1a,b, respectively clearly illustrates a decrease in vesicle size and an increase in the surface charge for concentration ≲20 mM, where MLVs have formed. The corresponding size polydispersity from DLS increases with increasing salt concentration as presented in Figure SM2, SI.

The SAXS data reveal how the interplay between the attractive and repulsive forces in the phospholipid bilayer determines the structure. Two parameters are particularly sensitive: the lamellar repeat distance, *d*, and the bilayer thickness, δ_HH_. With an increase in NaCl content the Cl^–^ and Na^+^ ions can associate with the trimethylammonium and phosphate groups of the polar lipid head, causing an effective decrease in the dipole potential of the PC lipid membrane.^44^ This causes an increase in electrostatic repulsion between the charged surfaces that overcomes the weak van der Waals attraction. These observations are supported by ∼3% swelling of δ_HH_, and ∼1% decrease in *d*, respectively for salt concentrations ≤10 mM. With further increase in NaCl content, the arrival of the new Cl^–^ ions starts to screen the existing electrostatic repulsion between the surfaces. This causes a sharp shrinkage of δ_HH_ by ∼5% and reduction of *d* by ∼13% at 470 mM.

Finally, we will compare the results presented here with earlier measurements of the membrane rigidity, κ_η_/*k*_B_*T*. Previous work studied the membrane rigidity for salt concentrations 0, 150, and 470 mM.^15^ The results depend on the analysis of the experimental data. However, an explicit trend shows (1) an increase by a factor of 1.5–2 from 0 to 150 mM, and (2) the values for 150 and 470 mM are the same within the experimental accuracy.^15^

Figure 1 shows that 150 and 470 mM are in region 3, corresponding to three bilayers. The initial value of 0 mM refers to the single bilayer. A higher κ_η_/*k*_B_*T* means a more stable liposome. Therefore, it is plausible to measure a higher κ_η_/*k*_B_*T*. The underlying reason has been introduced by Helfrich and discussed by Nagle and Tristram-Nagle.^45,46^ The membrane rigidity is connected with the molecular fluctuations of the lipids. More precisely, Zilman and Granek introduce the height–height correlation function.^47^ These undulations cause an effective interaction between two adjacent bilayers. Hence, a second bilayer suppresses independent fluctuations, equivalent to the observed increased bending rigidity. Decreasing the fluctuations decreases the entropy, increasing the Gibbs free energy, *F*. The minimum interbilayer water thickness, *t*_w,min_, can be reached for a rigid system that has only steric or excluded volume interactions, *F* ∝ (*k*_B_*T*)^2^*t*_w, min_^2^. For a flexible membrane at finite temperature, a modified term was suggested: *F* ∝ (*k*_B_*T*)^2^*t*_w, min_^2^ exp (−*t*_w_/λ). The empirically introduced decay constant, λ, was predicted to be connected with the decay length of the hydration force, λ_hyd_, by a factor of 2, λ = 2λ_hyd_.

The last two equations neglect osmotic pressure. Nagle and Tristram-Nagle display interbilayer water thickness and osmotic pressure.^45^ The thickness decreases from around 19 Å down to 5 Å within their osmotic pressure range. For our systems, the lowest concentration at which a bilayer forms is 8 mM, corresponding to an osmotic pressure of around 14 bar and an interbilayer water thickness of around 21 Å. Nagle and Tristram-Nagle obtain roughly 10 Å at this osmotic pressure, hence a factor of 2 lower.^45^ We measure approximately 15 Å at the highest concentration; Nagle and Tristram-Nagle report 7 Å at the equivalent.^45^

Despite the factor of 2, we observed a consistent decrease. The factor of 2 is not surprising because we compare two different systems and methods, and we used salt to increase the osmotic pressure. Within these differences, factor two is not surprising. Hence, we assume various interactions, including steric, hydration force, and fluctuations, cause the minimum interbilayer water thickness. We omit further details of the underlying energies and refer to the text by Nagle and Tristram-Nagle for a more detailed explanation of the theories.^45^ Finally, we return to the bending rigidity. We observed that the bending rigidity first decreased from 0 to 150 mM, but 150 and 470 mM were the same within the experimental accuracy. At first glance, this contradicts the increasing osmotic pressure. However, the constant value is consistent with observing a constant interbilayer water thickness for 150 and 470 mM.

## Conclusions

The study demonstrates how a seemingly simple and often overlooked environment with enhanced salt or ion concentration can be used to transform unilamellar to MLVs while controlling the overall size of the vesicle and water core simultaneously even at subphysiological concentrations. Our experimental results provide a plausible explanation other than the previously hypothesized balance between osmotic pressure and electrostatic interactions. One reason for the observation could be the experimental condition in which the salt was added after the self-assembly of the liposomes, i.e., only from the outside. Hence, initially (*t* = 0 s), salt can only be outside the membrane, not inside or in the inner water compartment. The experiments showed multiple transitioning stages of the neutral phospholipid vesicles with abrupt phase transitions at ϕ_M1_ = 8 mM, ϕ_M2_ = 75 mM, and ϕ_M3_ = 500 mM concentrations, which can be indicators for the formation of MLVs and higher-ordered hybrid structures in a saline environment window ranging from very low concentrations as in freshwater to very high as in water of the Dead Sea. Finding the continuous size change and abrupt phase transitions at specific concentrations are likely to enhance understanding of cell signaling, translocation, intracellular biological functioning, and cell division in the high saline environment but also will help design lipid drug delivery vesicles with controlled membrane transport properties with the strong dependence on the number of layers as a response to external salinity variation. This study focused on 0.25 wt % lipid concentration. Future work is required to identify the influence of the concentration.

Furthermore, since the permeation of molecules through layers is determined by the thickness and the number of layers, our results show that a change in the salt concentration is expected to affect the permeation rate. This will help to manipulate existing biocompatible materials and provide a better understanding of concentration-dependent permeation rates. For example, a novel pathway for controlling the encapsulation efficiency above 43% is demonstrated, which was achieved using different phospholipids.^48^

## Supporting Information Available

Data modeling; size distribution; normalized data in different presentations, lin-log, log–log, lin-lin; osmotic pressure; interbilayer water thickness vs concentration; and parameters obtained from SANS and SAXS (PDF)
