# Supplementary material for: Ion-Mediated Structural Discontinuities in Phospholipid Vesicles
Source: Langmuir. 2024 Jul 9;40(29):14990–5000. doi: 10.1021/acs.langmuir.4c01219 (PMC11270981; doi:10.1021/acs.langmuir.4c01219)
Supplement: Supplementary file 1 — la4c01219_si_001.pdf [file la4c01219_si_001.pdf]

Ion-Mediated Structural Discontinuities in Phospholipid Vesicles

Judith De Mel[1], Stefanie Klisch[1], Sudipta Gupta[1], and Gerald J. Schneider[1,2]
[1]*Department of Chemistry, Louisiana State University, Baton Rouge, LA 70803, USA*

[2]*Department of Physics & Astronomy, Louisiana State University, Baton Rouge, LA 70803, USA*

Supplementary Information

Data Modeling

Bilayer structure: The random lamellar sheet consisting of the heads and tails of the phospholipids can be modeled using the Caille structure factor. [S1, 2] It provides direct access to the macroscopic scattering cross-section given by the scattering intensity for a random distribution of the lamellar phase as

$$\frac{d\Sigma}{d\Omega}(Q) = 2\pi \frac{VP(Q)S(Q)}{Q^2 d} \tag{S1}$$

with the scattering volume, V, and the distance of the lamellae, d. The form factor is given by:

$$P(Q) = \frac{4}{Q^2}[\Delta\rho_{\mathrm{H}}\{\sin(Q(\delta_H + \delta_T)) - \sin(Q\delta_T)\} + \Delta\rho_{\mathrm{T}}\sin(Q\delta_T)]^2 \tag{S2}$$

The scattering contrasts for the head and tail are $\Delta\rho_{\mathrm{H}}$ and $\Delta\rho_{\mathrm{T}}$, respectively. The corresponding thicknesses are δ_H and δ_T, respectively, as presented in *Figure SM1.* The head-to-head bilayer thickness is given by, $\delta_{HH} = 2(\delta_H + \delta_T)$. The Caille structure factor is given by

$$S(Q) = 1 + 2\sum_{n=1}^{N-1}\left(1 - \frac{n}{N}\right)\cos(Qdn)\exp\left(-\frac{2Q^2 d^2 \alpha(n)}{2}\right) \tag{S3}$$

with the number of lamellar plates, N, and the correlation function for the lamellae, $\alpha(n)$, defined by

$$\alpha(n) = \frac{\eta_{\mathrm{cp}}}{4\pi^2}(\ln(\pi n) + \gamma_E) \tag{S4}$$

with γ_E = 0.57721 the Euler's constant. The elastic constant for the membranes are expressed in terms of the Caille parameter, $\eta_{\mathrm{cp}} = \frac{Q_0{}^2 k_B T}{8\pi\sqrt{\kappa_b\,\kappa_A}}$, where κ_b and κ_A are the bending elasticity and the compression modulus of the membranes, respectively. Here κ_A is associated with the interactions between the membranes. The position of the first-order Bragg peak is given by Q_0, and k_B is the Boltzmann's constant and *T* the absolute temperature.

Vesicle structure: The vesicle form factor is modeled using an extension of the core-shell model used in our previous studies.[S3, 4] The core is filled with water and in case of multilamellar liposomes encapsulated by *N* shells of lipids and *N*-1 layers of solvent as illustrated in *Figure SM1*. Each shell thickness and scattering length density is assumed to be constant for the respective shell. The 1D scattering pattern is described by:

$$P(Q,R,t,\Delta\rho) = \frac{\phi[F(Q)]^2}{V(R_N)} \tag{S5}$$

with

$$F(Q) = (\rho_{\mathrm{shell}} - \rho_{\mathrm{solv}}) \sum_{i=1}^{N} \left[3V(r_i) \frac{\sin(Qr_i) - Qr_i\cos(Qr_i)}{(Qr_i)^3} - 3V(R_i) \frac{\sin(QR_i) - Qr}{(QR_i)} \right. \tag{S6}$$

For

$$r_i = r_c + (i-1)(t_s + t_w) \quad R_i = r_i + t_s \qquad \begin{array}{l}\text{solvent radius before shell } i \\ \text{shell radius for shell } i\end{array} \tag{S7}$$

Here, *V(r)* is volume of the sphere with radius *r*, r_c is the radius of the core, t_s is the thickness of the individual shells, t_w is the thickness of the interleaved solvent layers, ϕ, the corresponding lipid volume fraction. For DOPC, we used the scattering length density of the shell, $\rho_{\mathrm{shell}} = 3.01 \times 10^9$ cm^{-2} and for D_2O the scattering length density of the solvent, $\rho_{\mathrm{solv}} = 6.36 \times 10^{10}$ cm^{-2}, respectively.[S5] The macroscopic scattering cross-section is obtained by

$$\frac{d\Sigma}{d\Omega}(Q) = \int dr P\big(Q,R,t,\rho_{\mathrm{lipo}},\rho_{\mathrm{solv}}\big) s(r) \tag{S8}$$

For the size polydispersity, $s(r)$, we used a Schulz distribution and a log-normal distribution. In addition, the thickness of the shell and the solvent are convoluted with a Gaussian distribution function to account for the thickness polydispersity.

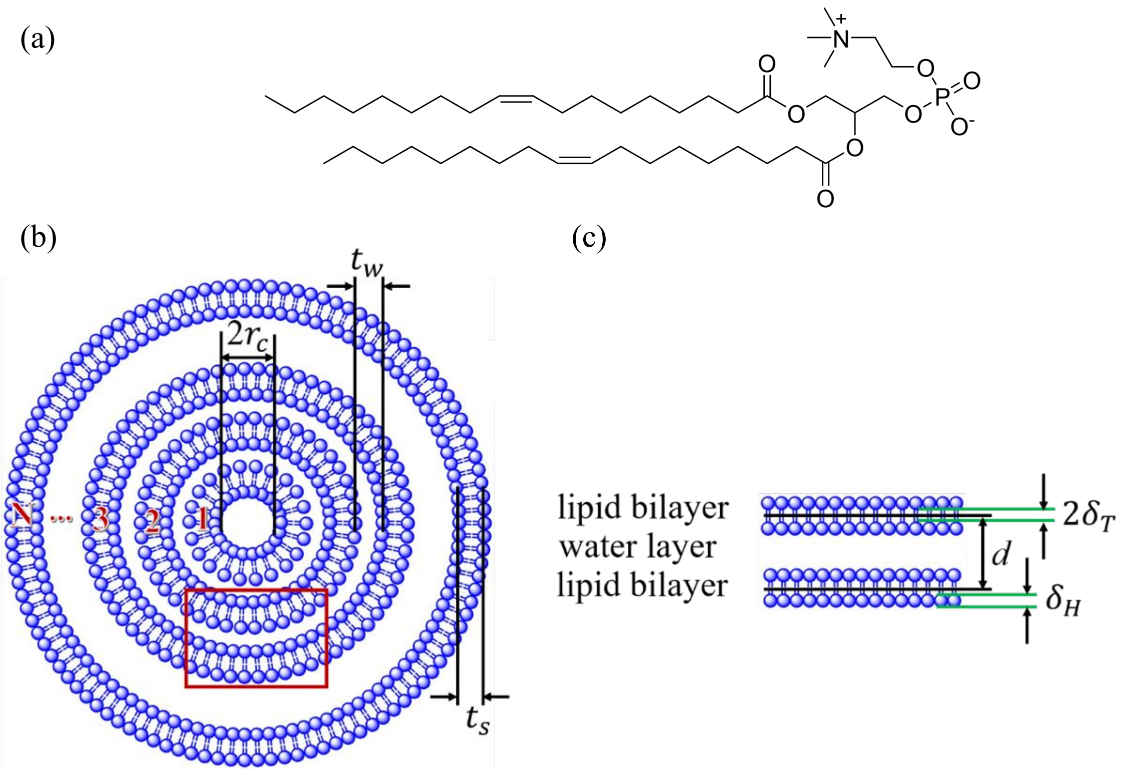

Figure SM1 – *Schematic representation of (a) the chemical structure, (b) the multilamellar liposome, and (c) lipid multilayers illustrating the number of bilayers, N, the radius of the core,* r_c*, the thickness of the individual shells,* t_s*, the thickness of the interleaved solvent layers,* t_w*, the thickness of the lipid head,* δ_H*, the thickness of the lipid tail region,* δ_T *and the lamellar repeat distance, d, of bilayers.*

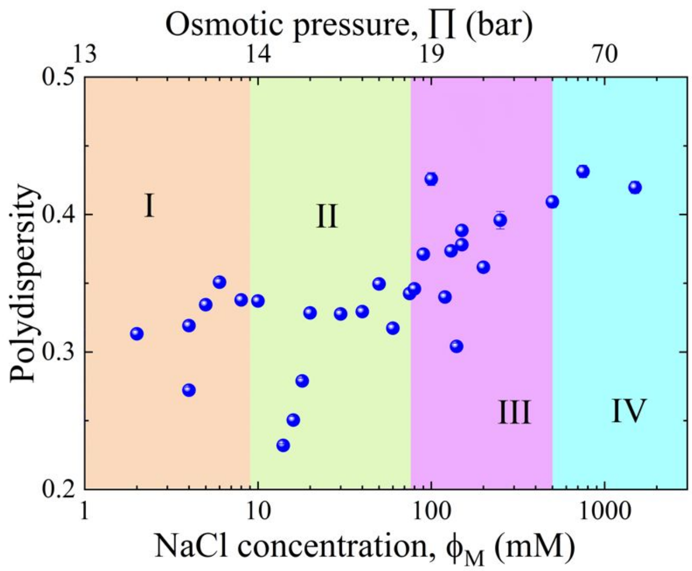

Figure SM2 – *Illustrating the size distribution of the hydrodynamic radius from DLS as a function of the salt concentration, ϕ_M and osmotic pressure,* Π.

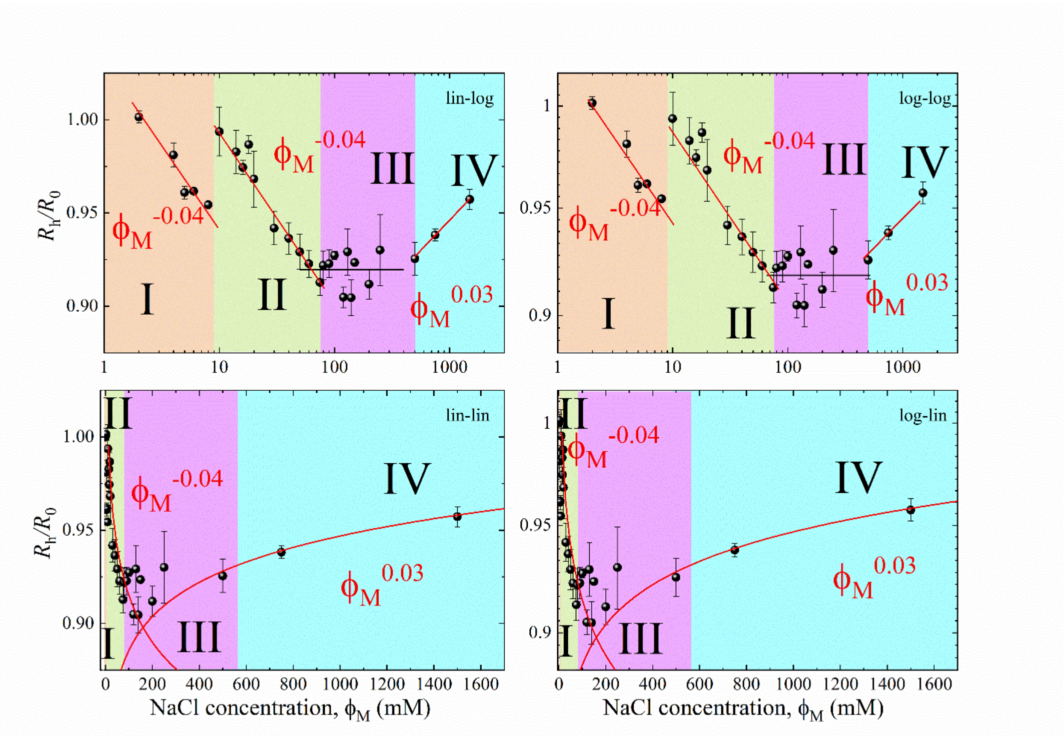

Figure SM3 – *The normalized hydrodynamic radius, R_h/R_0, as a function of the salt concentration, ϕ_M in lin-log, log-log, lin-lin and log-lin representations. Here R_0 is the hydrodynamic radius without any salt. The solid lines represent the different power laws. The four different regions are marked by different colors.*

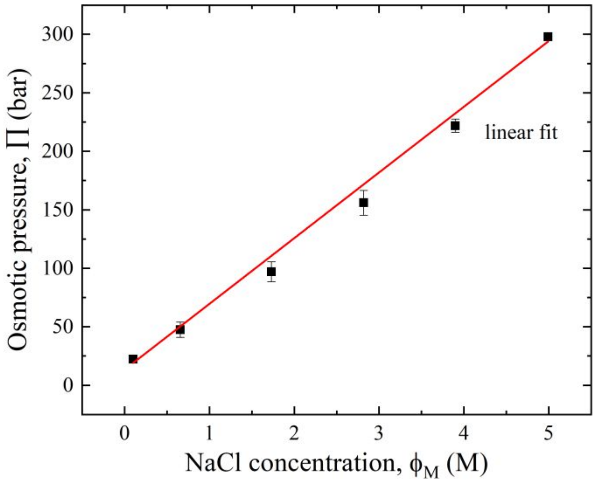

Figure SM4 – *Linear dependence of osmotic pressure, Π on NaCl concentration, ϕ_M.*[S6]

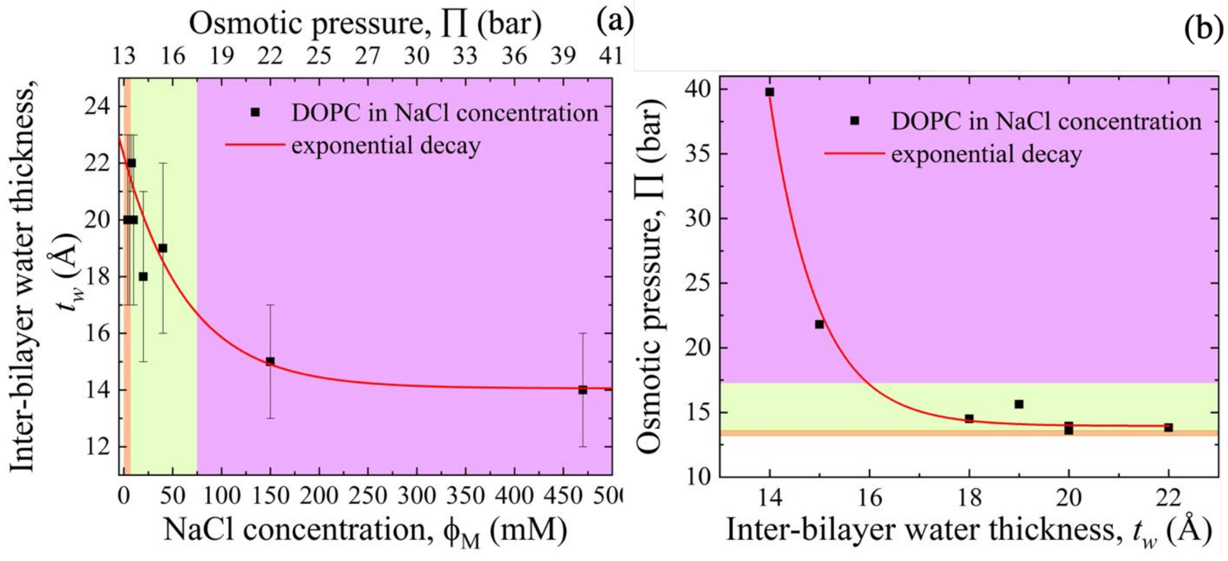

Figure SM5 – *(a) The lamellar repeat distance, d, as a function of the salt concentration, ϕ_M and the osmotic pressure, Π. The solid line represents an exponential decay, the same shape as for the interbilayer water layer thickness, t_w, illustrated in the main text. (b) Osmotic pressure, Π, as a function of the distance between the bilayers as given by the water layer thickness, t_w. The solid line is a fit assuming exponential decay for, $\Pi = P_0 exp\left(-t_w/\lambda\right)$, with the magnitude of the applied pressure, P_0, over a decay length, λ.*

Table S1 – Parameters obtained from SANS analysis, as illustrated in the main manuscript. For different salt concentration, $\boldsymbol{\phi_M}$, the number of layers in MLVs, *N*, the outer perimeter radius, R_{SANS}, and the thickness of the water layer between the bilayers $\boldsymbol{t_w}$.

ϕ_M (mM)	R_{SANS} (Å)	δ_{HH} (Å)	t_w (Å)	*N*
0	539 ± 8	39 ± 2	NA	1
2	530 ± 9	40 ± 2	NA	1
4	516 ± 11	38 ± 3	20 ± 2	2
6	498 ± 8	37 ± 2	20 ± 1	2
8	549 ± 10	38 ± 2	22 ± 1	2
10	539 ± 12	40 ± 2	20 ± 2	2
20	532 ± 11	42 ± 2	18 ± 2	2
40	525 ± 8	38 ± 2	19 ± 2	2
150	504 ± 8	39 ± 2	15 ± 2	3
470	502 ± 16	38 ± 2	14 ± 2	3

Table S2 – Parameters obtained SAXS analysis, as illustrated in Figure 4 of the main manuscript. For different salt concentration, $\boldsymbol{\phi_M}$, the number of layers, *N*, average lamellar spacing, *d*, size of the head, $\boldsymbol{\delta_H}$, the head-to-head bilayer thickness, $\boldsymbol{\delta_{HH}}$, the thickness of the water layer between the bilayers $\boldsymbol{t_w}$, and the Caille parameter, $\boldsymbol{\eta_{cp}}$.

ϕ_M (mM)	*N*	*d* (Å)	δ_H (Å)	δ_{HH} (Å)	t_w (Å)	η_{cp}
0	1	63 ± 1	6.7 ± 0.5	41.37 ± 1.2	NA	0.10 ± 0.01
6	3 ± 1	64 ± 1	7.1 ± 0.3	40.78 ± 2.2	22.70 ± 1.2	0.10 ± 0.01
10	3 ± 1	62 ± 1	7.8 ± 0.8	42.66 ± 3.0	20.00 ± 2.0	0.11 ± 0.02
20	3 ± 1	60 ± 2	7.8 ± 0.8	38.80 ± 1.0	21.20 ± 0.6	0.11 ± 0.01
40	3 ± 1	59 ± 1	6.1 ± 0.4	38.06 ± 1.2	20.57 ± 0.2	0.10 ± 0.01
150	4 ± 1	56 ± 1	6.0 ± 0.5	39.3 ± 1.4	16.85 ± 0.4	0.18 ± 0.02
470	4 ± 1	54 ± 1	6.0 ± 0.5	39.47 ± 1.2	15.06 ± 0.2	0.20 ± 0.02

References

S1. Nallet, F.; Laversanne, R.; Roux, D., Modelling X-ray or Neutron Scattering Spectra of Lyotropic Lamellar Phases: Interplay Between Form and Structure Factors. *Journal de Physique II* **1993,** *3*, 16.
S2. Berghausen, J.; Zipfel, J.; Lindner, P.; Richtering, W., Influence of Water-Soluble Polymers on the Shear-Induced Structure Formation in Lyotropic Lamellar Phases. *The Journal of Physical Chemistry B* **2001,** *105* (45), 11081-11088.
S3. Gupta, S.; Camargo, M.; Stellbrink, J.; Allgaier, J.; Radulescu, A.; Lindner, P.; Zaccarelli, E.; Likos, C. N.; Richter, D., Dynamic phase diagram of soft nanocolloids. *Nanoscale* **2015,** *7* (33), 13924-34.
S4. Gupta, S.; De Mel, J. U.; Perera, R. M.; Zolnierczuk, P.; Bleuel, M.; Faraone, A.; Schneider, G. J., Dynamics of Phospholipid Membranes beyond Thermal Undulations. *J Phys Chem Lett* **2018,** *9*, 2956-2960.
S5. Hoffmann, I.; Michel, R.; Sharp, M.; Holderer, O.; Appavou, M. S.; Polzer, F.; Farago, B.; Gradzielski, M., Softening of phospholipid membranes by the adhesion of silica nanoparticles - as seen by neutron spin-echo (NSE). *Nanoscale* **2014,** *6* (12), 6945-52.
S6. Luo, Y.; Roux, B., Simulation of Osmotic Pressure in Concentrated Aqueous Salt Solutions. *The Journal of Physical Chemistry Letters* **2009,** *1* (1), 183-189.
